# Nonsyndromic cleft lip and palate, gastric cancer and tooth agenesis

**DOI:** 10.4317/medoral.22132

**Published:** 2017-12-24

**Authors:** Eudes-Freire Cardoso, Daniella-Reis-Barbosa Martelli, Renato-Assis Machado, Ricardo D. Coletta, Júlia-Duarte de Souza, Fernanda-Tófani Barbosa, Maria-Fernanda-Leite de Figueiredo, Luiz-Gonzaga-Vaz Coelho, Hercílio Martelli-Júnior

**Affiliations:** 1 School of Medicine, State University of Montes Claros, Unimontes, Montes Claros, Minas Gerais, Brazil; 2 School of Dentistry, State University of Montes Claros, Unimontes, Montes Claros, Minas Gerais, Brazil; 3 Department of Oral Diagnosis, School of Dentistry, State University of Campinas, FOP-Unicamp, Piracicaba, São Paulo, Brazil; 4 Alfa Institute of Gastroenterology, Federal University of the State of Minas Gerais, UFMG, Belo Horizonte, Minas Gerais, Brazil; 5 Center for the Rehabilitation of Craniofacial Anomalies, University of José do Rosário Vellano, Unifenas, Alfenas, Minas Gerais, Brazil

## Abstract

**Background:**

To determine the frequency of nonsyndromic cleft lip and/or palate (NSCL/P) in first-degree relatives and to analyze the prevalence of tooth agenesis in patients with gastric cancer.

**Material and Methods:**

This cross-sectional, observational, case-control study included 798 patients attended at hospital Santa Casa in Montes Claros, Minas Gerais and Alfa Institute of Gastroenterology of the Federal University of the Minas Gerais. Information on basic demographic data and tooth agenesis of both groups and their family history of NSCL/P in first-degree relatives were evaluated. The collected information was stored in a database and analyzed using statistical program SPSS® version 21.0 and the values with *p*<0.05 were considered statistically significant.

**Results:**

Of the 798 patients, 113 (14.16%) consisted of the case group and 685 of the control group (85.84%). Non-Caucasian males were the most affected, although no differences among the groups were detected. Of all participants (n=798), 66 (8.27%) presented tooth agenesis and 25 (3.13%) presented oral cleft in first degree relative.

**Conclusions:**

Our results no found increase in the frequency of tooth agenesis in patients with gastric cancer and in the frequency of NSCL/P in the first-degree relatives of patients with gastric cancer.

** Key words:**Nonsyndromic cleft lip and/or palate, tooth agenesis, gastric cancer.

## Introduction

Nonsyndromic cleft lip and/or palate (NSCL/P) represents the most common orofacial birth defect, occurring in 1 in 500-2500 live births worldwide (1). In Brazil, the prevalence varies from 0.36 to 1.54 cases per 1000 live births (1,2). NSCL/P may be the result of a complex interplay between environmental exposures, genetic and epigenetic factors. Although in the past decade multiple genetic variants were associated with NSCL/P, providing valuable insights into its genetic etiology, the disease-susceptibility genes identified so far, only account for a small percentage of cases (3). Moreover, relatively few studies have investigated the association between genetic variants and environmental factors in NSCL/P (4).

It has been proposed that cancer and congenital malformations such as NSCL/P may occasionally have a common etiology. The underlying concept is that the same genes can act in both normal and malignant development (5,6). In the last years, epidemiological studies have assessed the relationship between cancer and NSCL/P in different populations (Texas, USA (7), France (8), Pittsburgh, USA (9), Latvia (6), Netherlands (10), Southeast Asian (11), and Brazil (12-14). Factors that have been suspected to be at the basis of these associations are polymorphic variants in genes involved in cell-to-cell adhesion and cell motility (6,15).

Cancer is a multifactorial disease in which both genetic and environmental factors play a significant role. Preventive plans for decreasing cancer incidence include both the removal of environmental agents known to be carcinogens and the identification of cancer susceptibility gene polymorphisms or mutations (16).

Gastric cancer is the fourth most common malignancy and the second leading cause of death due to cancer worldwide (17). In Brazil, the estimate for the year 2016, points to the occurrence of approximately 12,920 new cases of gastric cancer in men and 7,600 cases in women. These values correspond to an estimated risk of 13.19 new cases per 100,000 men and 7.41 cases per 100,000 women (18).

Studies have shown the relationship between cancer and dental anomalies (19,20), and other studies have shown the relationship between NSCL/P and dental anomalies (21,22). Hence, the aim of the current study is to determine the frequency of NSCL/P in first-degree relatives of patients with gastric cancer and to analyze the prevalence of tooth agenesis in patients with gastric cancer.

## Material and Methods

After proper approval of the State University of Montes Claros, Brazil, Institutional Review Board, 798 individuals were evaluated, 113 with gastric cancer were identified at the Oncology Clinic of the hospital Santa Casa, in the city of Montes Claros, Minas Gerais, and at the Alfa Institute of Gastroenterology of the Federal University of the State of Minas Gerais, in the city of Belo Horizonte, and 685 without gastric cancer or any syndrome were identified randomly at the General Clinics of Santa Casa in Montes Claros). All subjects from both groups (case and control) answered a questionnaire with questions about basic demographic information and their family history of NSCL/P in first-degree relatives (mother, father, son, daughter, siblings) and the evaluation of tooth agenesis was performed based on dental status and history. All permanent teeth were investigated, excluding third molars. The analyses focused on the type and number of missing teeth, the average number of missing teeth per patient. It was not identified any person with associated syndromes. The term hypodontia used to describe only a few missing teeth, while oligodontia refers to a more severe anomaly with six or more missing teeth (23).

The collected information was stored in a database and analysed using statistical program SPSS® version 21.0 (Statistical Package for Social Sciences for Windows®, Inc., USA). Statistical analyses were also carried out using Fisher’s exact test (expected frequency values fell below 5) and odds ratio (OR) with a confidence interval of 95% (95% CI) to estimate the magnitude of the risk. Values with *p*<0.05 were considered statistically significant.

## Results

From a total of 798 individuals of this study, 467 (58.53%) were female and 331 (41.47%) were male. In the case group, from the total of 113 patients, 67 (59.29%) were male and 46 (40.71%) female (*p*=0.000). The average age of the cases diagnosed with gastric cancer was 62.41 years (standard deviation ± 14.68 years). The average age of the unaffected individuals was 57.14 years (standard deviation ± 14.34 years). The age distribution was not different between affected and unaffected individuals (*p*=0.017) ( Table 1).

**Table 1:**
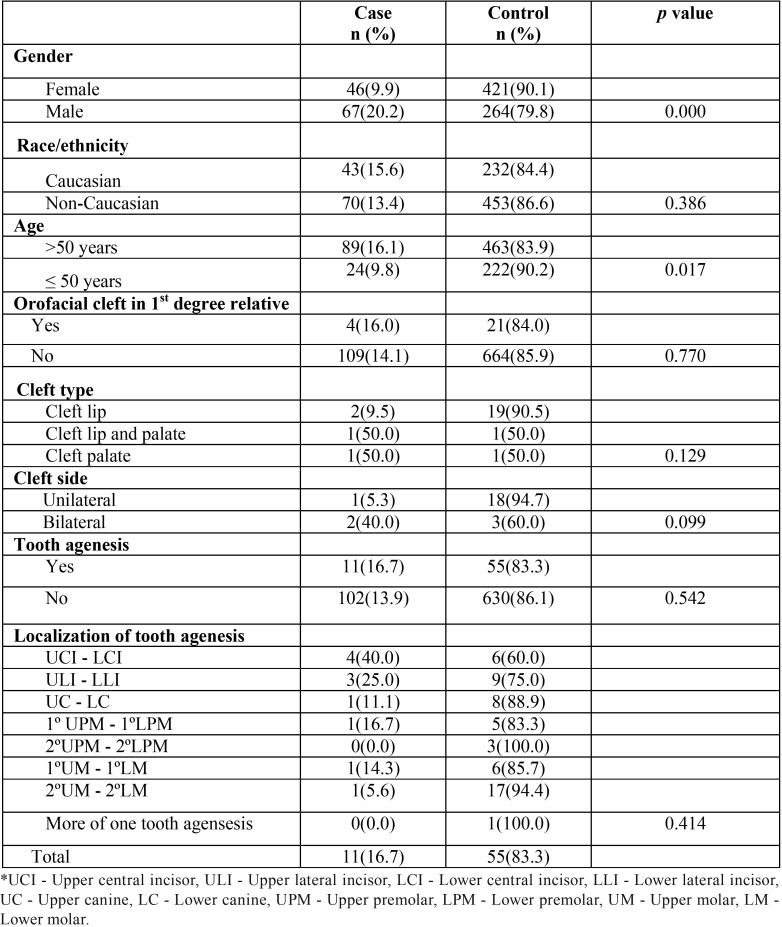
Characteristics of patients with gastric cancer and family history of Nonsyndromic cleft lip and/or palate.

From the 113 patients with gastric cancer, 4 (3.54%) had a positive history of NSCL/P, and in the control group, 21 individuals (3.06%) had a family history of NSCL/P (chi-square with 1 degree of freedom, *p*=0.77; CI 95%, 0.39–3.44). Regarding the presence of tooth agenesis between individuals of case and control group, it was found that in the first 11 patients with gastric cancer (9.73%) presented agenesis, while in the control group, 55 individuals (8.03%) presented the dental anomaly (chi-square with 1 degree of freedom, *p*=0.54; CI 95%, 0.62–2.43) ( Table 1). In all individuals of both groups, there were found only cases of hypodontia and no oligodontia. In patients with gastric cancer and in the control group there was a predominance of agenesis of maxillary lateral incisors. No general difference in the frequency of agenesis between the left and right sides was found.

In relation to ethnicity, in both groups there was a prevalence of non-Caucasians (case group: 61.94% versus 38.05% and control group: 66.13% versus 33.86%). The ancestry of individuals studied previously in the same place (Minas Gerais State, Brazil) was held (24). The average ancestry contributions to patients with NSCL/P were estimated as 87.5% European, 10.7% African, and 1.8% Amerindian. These results were similar to those of the control subjects (90% European, 7.5% African, and 2.5% Amerindian) (24).

## Discussion

Previous studies have shown that a high risk of oral clefts may exist in families where a cancer case has been identified (20,25). The increased occurrence of NSCL/P in hereditary diffuse gastric cancer (HDGC) patients with a *CDH1* mutation was suggested by Frebourg *et al.* (8) and supported by Kluijit *et al.* (10). Recently, the incorporation of a family history of orofacial cleft was suggested into the new HDGC-defining criteria (11). In a recent study, we suggest that polymorphic variants in *AXIN2* and *CDH1* may be associated with NSCL/P susceptibility, and reinforce the putative link between cancer and oral clefts (20).

However, in the present study, the frequency of NSCL/P was not significantly increased in the first-degree relatives of women with gastric cancer. These results agree with a recent study in which we show that the frequency of NSCL/P was not significantly increased in the first-degree relatives of women with breast cancer (13). In a systematic review of the literature, an increased risk of cancer among relatives of individuals with NSCL/P could not be entirely confirmed (15).

More than 300 genes are involved in odontogenesis, and mutations in several of these genes have been linked with hypodontia (26). The genes that control the development of teeth also have important functions in other organs and body systems (26). A Finnish family was described in which a nonsense mutation in *AXIN2* was found to co-segregate with an oligodontia phenotype (19).

The present study, involving patients with gastric cancer showed no increase in the frequency of dental agenesis, compared to patients without cancer. Our results are consistent with a study involving colorectal cancer and tooth agenesis (27). Bonds *et al.* (23) evaluated the relationship between ovarian cancer and tooth agenesis. Although the results did not show a direct relation between the two conditions, the authors proposed studies with broader populations. However, Küchler *et al.* (28) observed an increased frequency of familial history of breast cancer and prostate cancer in individuals with at least one missing premolar as well as an increased frequency of all cancers in the group with at least one missing upper lateral incisor. Chalothorn *et al.* (26) described an increased prevalence of hypodontia in women with epithelial ovarian cancer. Twenty percent of these women reported one or two missing teeth, versus three percent in a cancer-free control sample. These results were confirmed recently (29). The study shows a prevalence of hypodontia in 19.2% of women with epithelial ovarian cancer and in 6.7% of women in the control group.

The hypothesized association between cancer and NSCL/P could be attributed to factors that have been suspected to be at the basis of these associations, which are polymorphic variants in genes involved in cell-to-cell adhesion and cell motility (15). Limitations of our study were the relatively small number of patients with gastric cancer. Studies with larger samples and molecular analyses are needed to better understand the possible relationships in the etiology of cancer and NSCL/P.

## Conclusions

Our results suggest that the frequency of NSCL/P was not significantly increased in the first-degree relatives of women with gastric cancer. It was also observed that patients with gastric cancer showed no increase in the frequency of tooth agenesis, compared to patients without cancer.
